# Mortality Outcome Associated with Specific *KRAS*, *NRAS*, and *BRAF* Hot-Spot Mutations in Metastatic Colorectal Cancer Patients: A Retrospective Cohort Study

**DOI:** 10.3390/diagnostics15050590

**Published:** 2025-02-28

**Authors:** Omer Abdelgadir, Yong-Fang Kuo, M. Firoze Khan, Anthony O. Okorodudu, Yu-Wei Cheng, Jianli Dong

**Affiliations:** 1 Graduate School of Biomedical Science, University of Texas Medical Branch, Galveston, TX 77555, USA; 2 School of Public and Population Health, University of Texas Medical Branch, Galveston, TX 77555, USA; yokuo@utmb.edu; 3 Department of Pathology, University Texas Medical Branch, Galveston, TX 77555, USA; mfkhan@utmb.edu (M.F.K.); aookorod@utmb.edu (A.O.O.); 4 Department of Laboratory Medicine, Cleveland Clinic, Cleveland, OH 44195, USA; chengy@ccf.org

**Keywords:** *KRAS*, *NRAS*, *BRAF*, hot-spot mutation, mCRC, all-cause mortality, molecular biomarker, tumor biomarker, pyrosequencing

## Abstract

**Background/Objective:** The prognostic value of specific hot-spot mutations within *KRAS*, *NRAS*, and *BRAF* genes in metastatic colorectal cancer (mCRC) genes remains debatable. This study explores whether certain *KRAS*, *NRAS*, and *BRAF* mutations are associated with the risk of all-cause mortality in mCRC. **Methods:** We retrospectively analyzed records of 494 patients with mCRC treated at the University of Texas Medical Branch between January 2016 and July 2023. Data on genetic mutations and clinicopathological features were collected for this analysis. We estimated survival probabilities and conducted multivariable Cox proportional hazards regression to evaluate the impact of specific mutations on all-cause mortality risk. **Results:** *KRAS* c.35G>T (p.Gly12Val) and c.34G>T (p.Gly12Cys) mutations were significantly associated with an increased risk of all-cause mortality in the overall mCRC population and the treated mCRC subgroup. *KRAS* c.38G>A (p.Gly13Asp) was significantly associated with an increased risk of all-cause mortality in the treated mCRC subgroup but *BRAF* c.1799T>A (p.Val600Glu) was significantly associated with an increased risk of all-cause mortality in the overall mCRC population. No significant association was observed between *NRAS* mutations and mortality risk in mCRC, possibly due to their lower frequency or different biological effects compared to *KRAS* and *BRAF* mutations. **Conclusions:** These findings suggest that specific *KRAS* [c.35G>T (p.Gly12Val), c.34G>T (p.Gly12Cys), and c.38G>A (p.Gly13Asp)] and *BRAF* c.1799T>A (p.Val600Glu) mutations may have prognostic value in mCRC. However, given the single-center study design and lack of direct therapeutic implications, larger multicenter studies are needed to substantiate these results and better define the clinical relevance of these mutations.

## 1. Introduction

According to the American Cancer Society (ACS), colorectal cancer (CRC) is the third leading cause of cancer-related deaths in men and fourth in women. The ACS Cancer Statistics Center projects a total of 52,900 CRC-related deaths in the United States in 2025, including 28,900 deaths among males and 24,000 deaths among females [1]. Currently, the TNM stages, which measure tumor extent, lymph node involvement, and distant metastasis, is the only widely used prognostic tool for survival outcomes in CRC [2]. Köhne’s index is commonly utilized in advanced-stage cases [3].

The prognostic accuracy of TNM stages is limited by intra-tumoral heterogeneity within risk groups. While TNM stages can broadly categorize patients, both low-risk and high-risk groups can harbor significant biological diversity. This translates clinically, as some patients classified as low-risk may experience aggressive disease progression, while others designated high-risk may respond favorably to treatment, with minimal morbidity [4,5,6]. This limitation likely stems from the incomplete capture of underlying tumor biology by these markers. These markers do not fully account for the complex interplay of factors influencing prognosis, such as additional genetic alterations and unique tumor microenvironments. This observed heterogeneity within risk groups underscores the critical need for more precise risk stratification models.

Beyond TNM stages, emerging evidence suggests that factors such as the tumor’s genetic makeup and microenvironment may influence survival outcomes [7]. CRC progression is largely driven by the sustained activation of MAPK/ERK intracellular signaling pathways that regulate cell division and survival. Genetic alterations in RAS and BRAF often serve as key drivers, overriding normal regulatory mechanisms and fostering tumor growth [8]. Mutant *KRAS*, *NRAS*, and *BRAF* genes might increase the mortality risk by promoting uncontrolled cell proliferation and survival or enhancing invasion and metastasis [9]. Specifically, metastasis promotes cellular adaptation to new organ microenvironments, allowing cancer cells to evade the immune system and develop resistance to therapy. This also deprives healthy tissues by competing for nutrients and oxygen, disrupting vital organ function and potentially increasing the risk of mortality in CRC patients [10].

Existing research often classifies *KRAS*, *NRAS*, and *BRAF* mutations broadly as mutant versus wildtype [11]. However, this binary approach fails to capture the functional diversity of specific nucleotide changes within these genes. Different hot-spot mutations can lead to distinct alterations in protein activity, influencing intracellular signaling, tumor behavior, and disease progression. Some mutations may drive more aggressive phenotypes, impacting survival independently of treatment regimens [12]. Given these biological differences, understanding the prognostic impact of individual mutations is critical.

Overall survival is the most definitive, patient-centered, and universally accepted clinical endpoint in oncology [13,14]. While tumor shrinkage may suggest treatment efficacy, treatment response does not reliably predict survival benefits [15]. This is particularly relevant for *KRAS*, *NRAS*, and *BRAF* mutations, which drive resistance to anti-EGFR therapy. Relying solely on treatment response overlooks the broader impact of these mutations. Patients may initially respond but still experience poor long-term outcomes due to resistance or aggressive tumor biology. Put differently, treatment response does not equate to survival benefit. In this context, prioritizing treatment response over mortality risks misinterpreting the true prognostic impact of these mutations. Therefore, this study sought to investigate the association between specific hot-spot mutations within *KRAS*, *NRAS*, and *BRAF* genes and all-cause mortality among CRC patients with lymph node involvement and distant metastasis (mCRC). This analysis is imperative for identifying aggressive tumor behavior genetic markers and may offer an additional dimension in prognostic models.

## 2. Materials and Methods

### 2.1. Study Cohort

**Figure 1:**
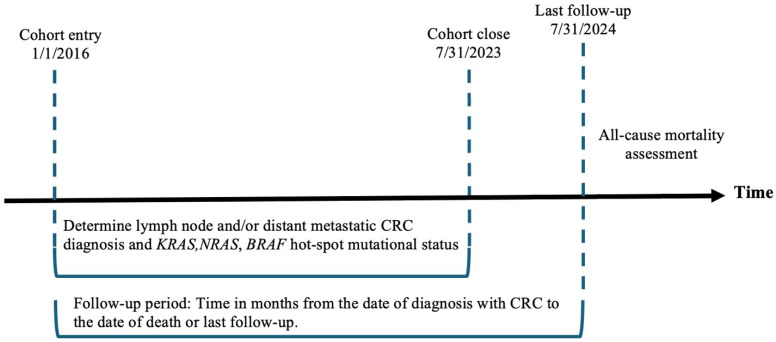
Study’s timeline.

A total of 494 patients with mCRC were included in this retrospective cohort study. Tumor samples from all patients were tested for *KRAS*, *NRAS*, and *BRAF* hot-spot mutations using pyrosequencing at the University of Texas Medical Branch (UTMB) between January 2016 and July 2023 (Figure 1). Demographics and clinicopathological data were obtained from patient health records. To ensure patient privacy, all data were de-identified. The Institutional Review Board at UTMB (Galveston, Texas, USA) approved the study under IRB protocol number 02-089, with approval granted on 10 June 2019.

### 2.2. Mutational Analysis

Tumor samples from mCRC patients, either from diagnostic biopsies or surgical resections, were analyzed for mutations. The QIAamp DNA formalin-fixed paraffin-embedded (FFPE) Tissue Kit (Qiagen, Germantown, MD, USA) was used for DNA extraction from FFPE tissues. *KRAS*, *NRAS*, and *BRAF* genes were amplified by PCR, targeting codons 12, 13, and 61 (*KRAS* and *NRAS*) and codon 600 (*BRAF*). The protocol included initial denaturation (95 °C, 15 min), 42 cycles of denaturation (95 °C, 20 s), annealing (53 °C, 30 s), and elongation (72 °C, 20 s), followed by a final elongation (72 °C, 5 min). Agarose gel electrophoresis was used to verify PCR products, and real-time pyrosequencing was used for mutation quantification with the PyroMark Q24 System [16].

### 2.3. Study Measures

The primary outcome was all-cause mortality. The study’s index date was defined as the date of initial CRC diagnosis within the study period (1 January 2016–1 August 2023). Patients were followed until 31 July 2024, to allow at least 12 months post-diagnosis follow-up time. The last follow-up date was the last encounter, as indicated in the patient’s chart. Patients who remained alive or died after the study’s end date were right-censored. Survival time was defined as the time in months from the date of CRC diagnosis to the date of death, last follow-up, or end of study, whichever came first. Three key predictors were analyzed: *KRAS* hot-spot mutations (categorized as wildtype, c.35G>A “p.Gly12Asp”, c.35G>T “p.Gly12Val”, c.34G>T “p.Gly12Cys”, c.38G>A “p.Gly13Asp”, and other variants); *NRAS* hot-spot mutations (grouped as wildtype, p.Gln61 mutations, other variants, and unknown status); and *BRAF* hot-spot mutation (categorized as wildtype, c.1799T>A “p.Val600Glu”, and unknown status). The potential confounders were selected based on a priori knowledge and included age at diagnosis, sex, race/ethnicity, family history of cancer, use of tobacco, number of comorbidities, primary tumor location, tumor grade, histopathological features, DNA mismatch repair (MMR), anemia, neutrophil-to-lymphocyte ratio (NLR), and carcinoembryonic antigen (CEA) levels. Additional details on the study variables are provided in Table S1.

### 2.4. Statistical Analysis

Patient characteristics were described using statistical summaries. Categorical data were presented as counts and percentages and compared using χ^2^ and Exact (Monte Carlo) tests where appropriate. Numerical variables were reported as means with standard deviations (SDs) and medians with interquartile ranges (IQRs) and compared using ANOVA. Overall survival (OS) probabilities were estimated using a non-parametric Kaplan–Meier method, and differences between *KRAS*, *NRAS*, and *BRAF* groups were assessed using log-rank tests. Potential interactions between *KRAS*, *NRAS*, and *BRAF* with the other covariates were evaluated prior to final model selection, and no significant interaction was observed.

Multivariable Cox proportional hazard regressions were conducted to estimate the hazard ratio (HR) of all-cause mortality. The Exact method was used to handle ties in the Cox models. To account for the potential moderation effect of treatment modalities on the risk of all-cause mortality, we conducted multivariable Cox models restricted to the patients who received either curative surgery, chemotherapy, or radiotherapy, whether as a single treatment or in combination. Additional analyses were performed with the stepwise selection method based on Akaike Information Criterion (AIC) reduction for parsimonious multivariable Cox models. Log–log survival plots, smoothed hazard plots, and weighted Schoenfeld residuals were utilized to check the proportional hazards assumption, and no violation was observed. Statistical significance was defined as a *p*-value < 0.05. Data analysis was performed using SAS software (version 9.4, SAS Institute, Cary, NC, USA) and R software (RStudio, version 4.4.1, Boston, MA, USA).

## 3. Results

In this retrospective study, we analyzed previously obtained pyrosequencing data of hot-spot mutations in the *KRAS*, *NRAS*, and *BRAF* genes from 494 patients with mCRC. Descriptive analysis was performed to characterize the mutation patterns. Table 1 summarizes the hot-spot mutations within *KRAS*, *NRAS*, and *BRAF* genes. For the *KRAS* gene, 41.8% were mutated. The most common mutations were identified in exon 2 codon 12, with c.35G>A (p.Gly12Asp) and c.35G>T (p.Gly12Val) occurring in 14.9% and 10.3% of cases, respectively. Another notable mutation included c.38G>A (p.Gly13Asp) at 7.7%. In contrast, the *NRAS* gene exhibited a lower mutation rate than the *KRAS* gene, with only 4.8% of mutated cases. The most frequent *NRAS* mutation was p.Gln61Lys (Q61K), detected in 1.8% of samples. For *BRAF*, a similar pattern of low mutation rate emerged, with c.1799T>A (p.Val600Glu) mutation observed in 6.9% of cases.

Table 2 presents the characteristics of patients with mCRC according to *KRAS*, *NRAS*, and *BRAF* genes’ mutational status. The patient population consisted primarily of male patients (67.6%), with a mean age of 61 years for the entire cohort. Notably, patients with the *BRAF* mutation were significantly older, with a mean age of 70.9 years, compared to 60.5 years for the wildtype, 59.9 years for the *KRAS* mutation, and 61.7 years for the *NRAS* mutation groups. Approximately half (47.5%) of the mCRC patients had wildtype genotypes across all three genes. *KRAS* mutations were the most prevalent, observed in 41.8% of cases. *NRAS* and *BRAF* mutations were less frequent, occurring in 4.8% and 6.9% of patients. The majority of *KRAS* and *BRAF* mutations were detected in right-sided tumors, accounting for 38.1% and 67.7% of cases, respectively. In contrast, *NRAS* mutations were more commonly found in left-sided tumors, with a prevalence of 52.2%. Patients with *BRAF* mutations demonstrated a higher frequency of deficient DNA MMR, as well as high-grade (G3) and mucinous-type tumors, with rates of 58.8%, 32.2%, and 35.3%, respectively. Moreover, elevated CEA levels were more commonly observed among patients with *KRAS* mutations (73.8%) compared to those in the wildtype, *NRAS* mutation, and *BRAF* mutation groups. Patients with *KRAS* mutations exhibited a higher rate of overall distant organ metastasis (70.3%), including metastasis to the liver (44.1%) and lungs (19.8%), whereas patients with *BRAF* mutations showed a higher rate of lymph node metastasis (82.4%). Of note, two patients’ mCRC exhibited concurrent *KRAS* and *NRAS* mutations. The first patient was male with left-sided CRC adenocarcinoma and synchronous liver metastases and harbored *KRAS* c.35G>T (p.Gly12Val) and *NRAS* c.181C>A (p.Gln61Lys) mutations. The second patient was female with rectal mucinous adenocarcinoma and synchronous bone metastases and presented with *KRAS* c.35G>T (p.Gly12Val) and *NRAS* c.38G>T (p.Gly13Val) mutations. Both cases demonstrated microsatellite stable tumors. Further descriptive analyses for patients’ characteristics according to specific hot-spot mutations in *KRAS*, *NRAS*, and *BRAF* genes are presented in Tables S2–S4.

**Figure 2:**
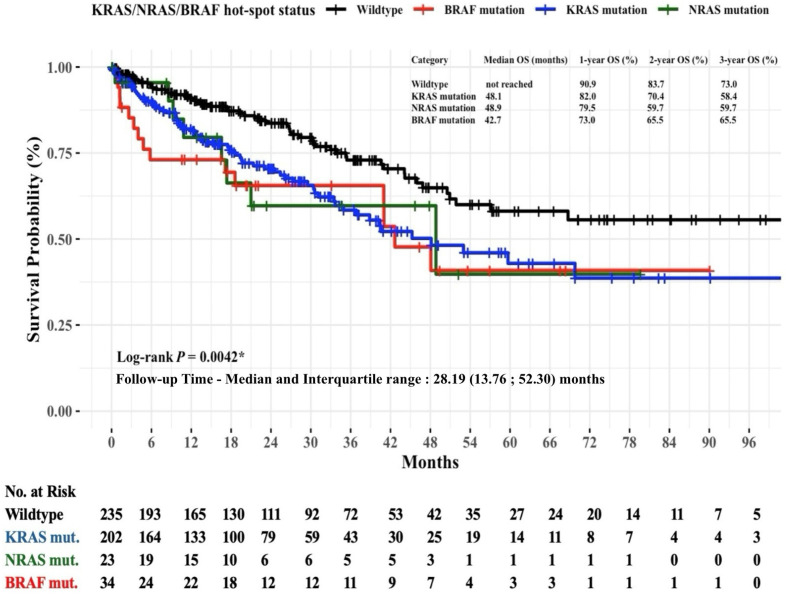
Non-parametric Kaplan–Meier estimates of time to death for patients with mCRC stratified by *KRAS*, *NRAS*, and *BRAF* hot-spot mutations (*n* = 494). Abbreviations: *BRAF*, v-raf murine sarcoma viral oncogene homolog B1; *KRAS*, Kirsten rat sarcoma viral oncogene homolog; mut, mutation; *NRAS*, neuroblastoma RAS viral oncogene homolog; OS, overall survival. * Statistical significance at the *p*-value < 0.05 level.

Non-parametric Kaplan–Meier estimator was used for OS probability analysis in mCRC patients stratified by the mutational status of *KRAS*, *NRAS*, and *BRAF* hot-spots. In Figure 2, the survival curve for the wildtype group demonstrated a clear survival benefit compared to the mutation groups. The median OS for the wildtype group could not be calculated due to the high proportion of patients (exceeding 50%) who remained alive at the last observed event time. The median OS for patients with *KRAS*, *NRAS*, and *BRAF* mutations was 48.1 months, 48.9 months, and 42.7 months, respectively. The OS rates significantly differed between mutation groups (log-rank *p* = 0.0042). At the three-year follow-up mark, the OS rates were 73% for the wildtype group, 58.4% for the *KRAS* mutation group, 59.7% for the *NRAS* mutation group, and 65.5% for the *BRAF* mutation group.

**Figure 3:**
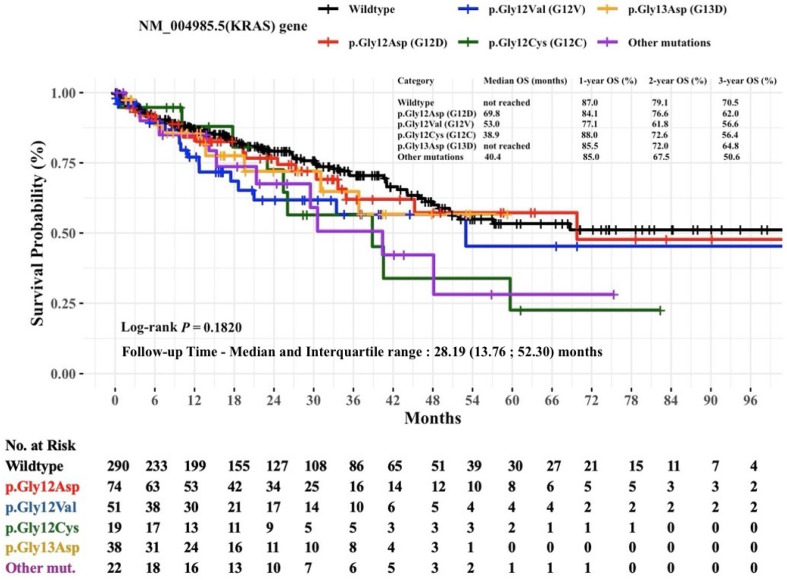
Non-parametric Kaplan–Meier estimates of time to death for patients with mCRC stratified by NM_004985.5(*KRAS*) hot-spot mutations (*n* = 494). Abbreviations: *KRAS*, Kirsten rat sarcoma viral oncogene homolog; mut, mutation; OS, overall survival. Other *KRAS* mutations include p.Gly12Ser (G12S), p.Gly12Ala (G12A), p.Gly12Arg (G12R), p.Gln61His (Q61H), p.Gln61Leu (Q61L), p.Gln61Arg (Q61R), and p.Gln61Glu (Q61E).

Figure 3 displays Kaplan–Meier estimates of the OS in mCRC patients stratified by specific hot-spot mutations in the *KRAS* gene. The median OS for the wildtype and c.38G>A (p.Gly13Asp) groups could not be calculated due to the high proportion of patients (exceeding 50%) who remained alive at the last observed event time. The group with the shortest median OS was the c.34G>T (p.Gly12Cys) mutation, exhibiting a median OS of 38.9 months. In contrast, the c.35G>A (p.Gly12Asp) and c.35G>T (p.Gly12Val) groups demonstrated a median OS of 69.8 months and 53.0 months, respectively. However, the difference in OS rates among the *KRAS* mutation groups was not statistically significant (log-rank *p* = 0.1820).

**Figure 4:**
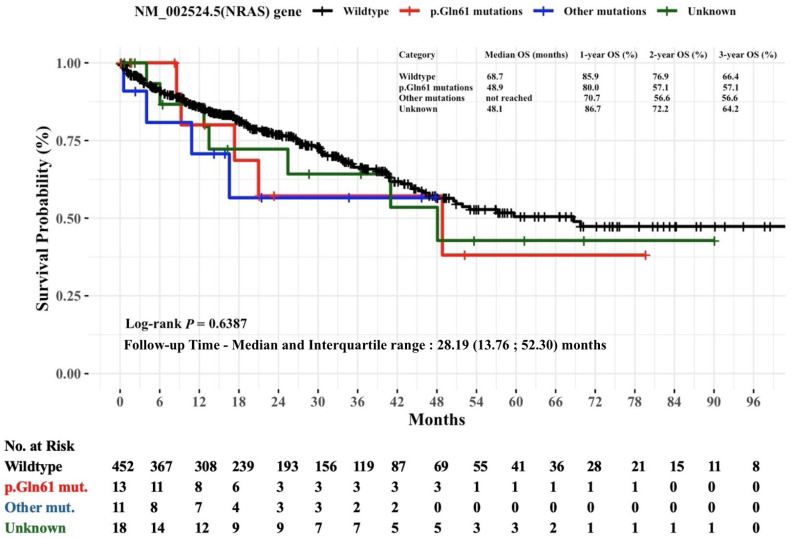
Non-parametric Kaplan–Meier estimates of time to death for patients with mCRC stratified by NM_002524.5(*NRAS*) hot-spot mutations (*n* = 494). Abbreviations: mut, mutation; *NRAS*, neuroblastoma RAS viral oncogene homolog; OS, overall survival. The *NRAS* p.Gln61 mutations include p.Gln61Lys (Q61K), p.Gln61Arg (Q61R), p.Gln61His (Q61H), and p.Gln61Leu (Q61L). Other *NRAS* mutations include c.35G>A (p.Gly12Asp), c.35G>T (p.Gly12Val), c.34G>T (p.Gly12Cys), p.Gly12Ser (G12S), and p.Gly13Val (G13V).

Figure 4 shows Kaplan–Meier estimates of the OS in mCRC patients stratified by specific hot-spot mutations in the *NRAS* gene. Patients with the wildtype *NRAS* gene had an OS of 68.7 months. While the p.Gln61 mutations group had 48.9 months, the other mutations group did not reach the median OS. At the three-year follow-up mark, the OS rates were relatively similar in patients with the *NRAS* mutation, as the p.Gln61 mutations group had a 57.1% OS rate and the other mutations group had a 56.6% OS rate. Overall, the difference in OS rates between *NRAS* mutation groups was not statistically significant (log-rank *p* = 0.6387).

**Figure 5:**
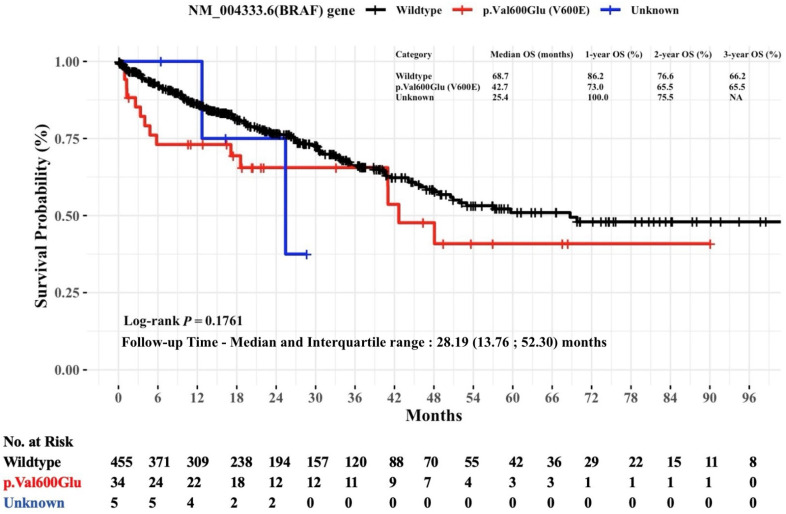
Non-parametric Kaplan–Meier estimates of time to death for patients with mCRC stratified by NM_004333.6(*BRAF*) hot-spot mutations (*n* = 494). Abbreviations: *BRAF*, v-raf murine sarcoma viral oncogene homolog B1; mut, mutation; OS, overall survival.

Figure 5 illustrates Kaplan–Meier estimates of the OS in mCRC patients stratified by specific hot-spot mutations in the *BRAF* gene. While patients with the wildtype *BRAF* gene had a median OS of 68.7 months, patients with the c.1799T>A (p.Val600Glu) mutation had a median OS of 42.7 months. Both groups had relatively similar OS rates at the three-year follow-up mark, as the wildtype group had a 66.2% OS rate and the c.1799T>A (p.Val600Glu) mutation had a 66.5% OS rate. The difference in OS rates between *BRAF* mutation groups was not statistically significant (log-rank *p* = 0.1761).

**Figure 6:**
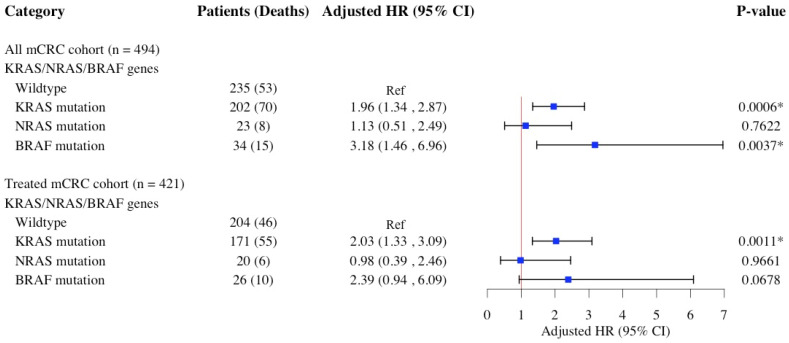
HRs and 95% CIs for the association of *KRAS*, *NRAF*, and *BRAF* hot-spot mutations and all-cause mortality risk in patients with mCRC. Multivariable Cox models adjusted for age at diagnosis, sex, race/ethnicity, primary tumor site, DNA mismatch repair status, tumor grade, tumor histomorphology, familial risk, tobacco use, comorbidities, anemia, neutrophil–lymphocyte ratio, and carcinoembryonic antigen. The treated mCRC cohort included patients who received curative surgery, chemotherapy, or radiotherapy, whether as a single treatment or in combination. Abbreviations: *BRAF*, v-raf murine sarcoma viral oncogene homolog B1; CI, confidence interval; HR, hazard ratio; *KRAS*, Kirsten rat sarcoma viral oncogene homolog; *NRAS*, neuroblastoma RAS viral oncogene homolog. * Statistical significance at the *p*-value < 0.05 level.

Figure 6 presents the results of multivariable Cox proportional hazards regressions assessing the association between *KRAS*, *NRAS*, and *BRAF* hot-spot mutations and the risk of all-cause mortality in mCRC patients. Compared to the wildtype group, both *KRAS* and *BRAF* mutations were significantly associated with an increased risk of all-cause mortality (*KRAS*: aHR: 1.96; 95% CI: 1.34–2.87; *p*: 0.0006 and *BRAF*: aHR: 3.18; 95% CI: 1.46–6.96; *p*: 0.0037) in overall mCRC population. The increased risk associated with *KRAS* mutation persisted in the treated mCRC subgroup (aHR: 2.03; 95% CI: 1.33–3.09; *p*: 0.0011), while the association between *BRAF* mutation and risk of all-cause mortality was not significant in this subgroup (*p* = 0.0678). No significant association was observed between *NRAS* mutation and the risk of all-cause mortality in either the overall mCRC population or the mCRC-treated subgroup. In a subsequent analysis, multivariable Cox proportional hazards regression was performed with a reduced set of covariates, selected according to the principle of parsimony and optimized by the AIC. Stepwise selection was applied with a *p*-value threshold of 0.2 for entry. The results from these optimized models, presented in Figure S1, are consistent with those shown in Figure 6, further reinforcing the robustness of the study’s findings.

**Figure 7:**
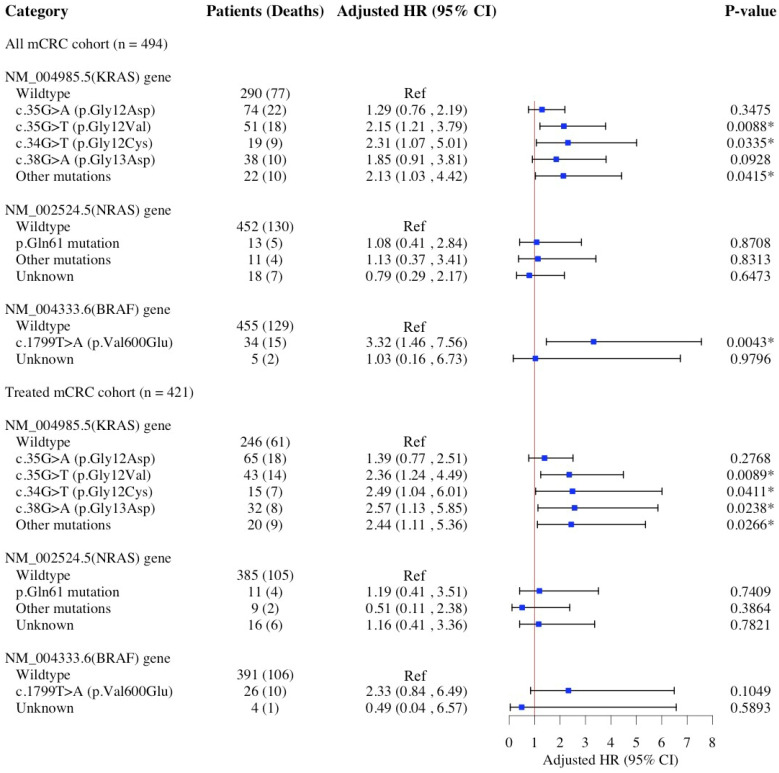
HRs and 95% CIs for the association of specific *KRAS*, *NRAF*, and *BRAF* hot-spot mutations and all-cause mortality risk in patients with mCRC. Multivariable Cox models adjusted for age at diagnosis, sex, race/ethnicity, primary tumor site, DNA mismatch repair status, tumor grade, tumor histomorphology, familial risk, tobacco use, comorbidities, anemia, neutrophil–lymphocyte ratio, and carcinoembryonic antigen. The treated mCRC cohort included patients who received curative surgery, chemotherapy, or radiotherapy, whether as a single treatment or in combination. Abbreviations: *BRAF*, v-raf murine sarcoma viral oncogene homolog B1; CI, confidence interval; HR, hazard ratio; *KRAS*, Kirsten rat sarcoma viral oncogene homolog; *NRAS*, neuroblastoma RAS viral oncogene homolog. * Statistical significance at the *p*-value < 0.05 level.

Figure 7 displays the results of multivariable Cox proportional hazards regressions examining the association between specific hot-spot mutations in *KRAS*, *NRAS*, and *BRAF* genes and the risk of all-cause mortality in mCRC patients. The *KRAS* c.35G>T (p.Gly12Val) mutation, as opposed to the *KRAS* wildtype, was associated with an increased risk of all-cause mortality (aHR: 2.15; 95% CI: 1.21–3.79; *p*: 0.0088) in the overall mCRC population, and this association persisted in the treated mCRC subgroup (aHR: 2.36; 95% CI: 1.24–4.49; *p*: 0.0089). Similarly, the *KRAS* c.34G>T (p.Gly12Cys) mutation was associated with an increased risk of all-cause mortality in both the overall mCRC population (aHR: 2.31; 95% CI: 1.07–5.01; *p*: 0.0335) and the treated mCRC subgroup (aHR: 2.49; 95% CI: 1.04–6.01; *p*: 0.0411). In addition, *KRAS* c.38G>A (p.Gly13Asp) was associated with an increased risk of all-cause mortality among the treated mCRC (aHR: 2.57; 95% CI: 1.13–5.85; *p*: 0.0238), but not the overall mCRC, population. The *BRAF* c.1799T>A (p.Val600Glu) mutation, as opposed to *BRAF* wildtype, was associated with an increased risk of all-cause mortality (aHR: 3.32; 95% CI: 1.46–7.56; *p*: 0.0043) in the overall mCRC population. However, this association was not maintained in the treated mCRC subgroup (*p*: 0.1049). No significant association was observed between *NRAS* mutations and the risk of all-cause mortality in either the overall mCRC population or the treated mCRC subgroup. We performed a more parsimonious multivariable Cox proportional hazards regression, selecting covariates through stepwise AIC with a *p*-value threshold of 0.2 (Figure S2). The results from these optimized models are consistent with those presented in Figure 7.

## 4. Discussion

Our study assessed the association between specific hot-spot mutations within *KRAS*, *NRAS*, and *BRAF* genes and all-cause mortality in mCRC patients. Overall, *KRAS* mutations were more prevalent compared to *NRAS* and *BRAF* mutations, highlighting the predominant role of *KRAS* in CRC tumorigenesis. Patients with mutated *KRAS*, *NRAS*, and *BRAF* genes demonstrated poorer overall survival compared to those with the wildtype genotype across all three genes. When these genes were further categorized by specific hot-spot mutations, *KRAS* c.35G>T (p.Gly12Val), c.34G>T (p.Gly12Cys), and c.38G>A (p.Gly13Asp), as well as *BRAF* c.1799T>A (p.Val600Glu), were independently associated with an increased risk of all-cause mortality in mCRC patients.

Although the *KRAS* gene has a powerful predictive utility for responses to anti-EGFR monoclonal antibody therapy, its prognostic value in CRC has yet to be determined [17,18,19,20]. Therefore, most professional agencies do not support utilizing *RAS* mutational status for prognostic purposes [11]. Consistent with previous studies [21,22,23,24,25,26,27], our analysis of mCRC patients demonstrates poorer prognosis associated with *KRAS* c.35G>T (p.Gly12Val) and c.34G>T (p.Gly12Cys) mutations compared to the *KRAS* wildtype. This finding holds true and extends to include c.38G>A (p.Gly13Asp) in the treated mCRC subgroup. A preclinical study has revealed distinct differences in GTP hydrolysis among various *KRAS* mutation subgroups influenced by intrinsic factors and GTPase-activating proteins. This variation leads to the differential activation of downstream pathways, particularly the MAPK/ERK signaling pathway. Notably, *KRAS* mutants like c.35G>A (p.Gly12Asp), c.34G>T (p.Gly12Cys), and *KRAS* c.38G>A (p.Gly13Asp) exhibit higher intrinsic GTPase activity compared to mutations, indicating the biochemical diversity across *KRAS* mutations [28,29]. These findings suggest caution in combining *KRAS* mutations from different exons/codons in biomarker assessments and clinical trials, highlighting the need for further validation studies. Overall, the study indicates that different hot-spot mutations may be associated with varying biological behaviors and potentially distinct prognostic and clinical outcomes.

In line with our study, previous studies have recognized the *KRAS* c.35G>T (p.Gly12Val) mutation as an independent prognostic factor linked to poorer survival outcomes [30,31]. In preclinical models, the *KRAS* c.35G>T (p.Gly12Val) mutation was associated with increased cell survival, invasion, and intravasation, correlating with the aggressive behavior seen in mCRC patients [32]. This mutation, similar to other *KRAS* mutations, activates the KRAS protein constitutively, driving downstream pathways like MAPK and PI3K/AKT, which promote unchecked cell proliferation [33,34]. Compared to *KRAS* wildtype, c.35G>T (p.Gly12Val) deregulates genes related to the cell cycle and apoptosis, reducing apoptosis and enhancing angiogenesis via IL8 [35]. This mutation was also linked to higher metastatic efficiency, as evidenced by increased tumor budding, higher CXCR4 expression, and enhanced Akt activation, which collectively contribute to increased cell survival, invasion, and intravasation [32]. Furthermore, the *KRAS* c.35G>T (p.Gly12Val) mutation was associated with a poor immune response within the tumor microenvironment, characterized by lower tumor-infiltrating lymphocytes and higher tumor–stromal percentage, which exacerbates the aggressive nature of *KRAS* c.35G>T (p.Gly12Val) in mCRC [36].

Similar to our study findings, a multicenter cohort study has shown poorer survival outcomes associated with *KRAS* c.34G>T (p.Gly12Cys) compared to *KRAS* wildtype [28]. This is likely due to its potential role in promoting resistance to chemotherapy and targeted therapy, as evidenced by the increased resistance to *KRAS* G12C inhibitors, such as sotorasib and adagrasib, which have shown limited efficacy in CRC compared to non-small cell lung cancer (NSCLC) [37,38]. In addition, similar to KRAS c.35G>T (p.Gly12Val), c.34G>T (p.Gly12Cys) has been associated with reduced tumor-infiltrating lymphocytes and an increased tumor–stromal percentage [34]. Furthermore, KRAS c.34G>T (p.Gly12Cys) presents unique challenges in treatment, potentially promoting negative feedback through the EGFR receptor, which may require combination therapies with anti-EGFR monoclonal antibodies to improve treatment efficacy [39].

The *KRAS* c.38G>A (p.Gly13Asp) mutation represents a distinct subgroup of CRC with unique biological characteristics and potential treatment implications. The computational study utilized molecular dynamics simulations, and the potential of mean force simulations has revealed that the *KRAS* c.38G>A (p.Gly13Asp) mutation exhibits unique structural and dynamic properties compared to other *KRAS* mutations. Specifically, the GTP-binding pocket in c.38G>A (p.Gly13Asp) is less accessible, potentially impacting protein stability and GTP interactions [40]. Moreover, *KRAS* c.38G>A (p.Gly13Asp) mutations activate a specific signaling pathway associated with liver metastasis involving the MEK-Sp1-DNMT1-miR-137-YB-1-IGF-IR pathway. This pathway is regulated by various factors, including IGF-IR activation, highlighting the complex interplay of signaling pathways in tumor progression [41]. Unlike other *KRAS* mutations, c.38G>A (p.Gly13Asp) retains a degree of sensitivity to neurofibromin-mediated GTP hydrolysis. This suggests that tumors harboring *KRAS* c.38G>A (p.Gly13Asp) might be more responsive to anti-EGFR monoclonal antibody therapy [42]. These distinctions may help explain the unique biological behavior and varied survival outcomes observed in patients with mCRC who have the *KRAS* c.38G>A (p.Gly13Asp) mutation in their tumors.

The *BRAF* c.1799T>A (p.Val600Glu), in addition to repressing the pro-apoptotic Bcl-2-like protein 11 (BIM) [43], induces a distinct DNA damage response that can impair p53 function, contributing to chemotherapeutic resistance [44]. Moreover, this mutation stimulates AMP-activated protein kinase (AMPK)-mediated autophagy, a cytoprotective mechanism that can complicate treatment outcomes [45]. The *BRAF* inhibition in CRC often leads to the upregulation of receptor tyrosine kinases (RTKs) and their downstream effectors, such as Gab2, which can activate alternative survival pathways and contribute to resistance against *BRAF* inhibitors [46]. This resistance is often mediated by mechanisms such as RAF dimerization, which can be targeted by the combined inhibition of both EGFR and RAF dimers [47].

The PETACC-3 trial showed that those who carry the *BRAF* c.1799T>A (p.Val600Glu) mutation and MSI-Low have an increased hazard of death as compared to those with wildtype *BRAF* [48]. Similar to our study, the CRYSTAL trial, the *BRAF* c.1799T>A (p.Val600Glu) mutation was significantly associated with a worse prognosis than wildtype *BRAF* [49]. While this observation raises questions about potential treatment-related modifications of the mortality risk, these findings should be interpreted with caution, as multiple factors such as differences in treatment modalities, patient selection, and unmeasured genetic co-alterations could contribute to the observed outcomes. Moreover, the findings underscore the importance of stratified analysis by treatment modality in prognostic studies, given the potential for treatment-related effect modification.

The prognostic significance of *NRAS* hot-spot mutations in mCRC remains a topic of debate. Some studies have indicated that mCRC patients with *NRAS*-mutated tumors experience a poorer prognosis [50,51]. In contrast, a pooled analysis of five randomized controlled trials (RCTs) found no significant prognostic value associated with *NRAS* mutations, aligning with our study’s findings [25]. This apparent discrepancy in the prognostic impact of *NRAS* mutations is likely due to their low frequency in mCRC or their subtle effect sizes, which may necessitate larger-scale studies for the definitive assessment of their prognostic role in mCRC.

The strength of our study lies in its use of real-world data, which provides a more accurate representation of current clinical practices beyond the controlled environment of clinical trials. Additionally, our consistent application of pyrosequencing for tumor genotyping enhances the internal validity of our study. Analyzing *KRAS*, *NRAS*, and *BRAF* hot-spot mutations at both the nucleotide and protein levels may offer insights into the mutational processes that influence tumor behavior, but functional studies are needed to validate these observations. We also performed subgroup analyses to address how standard mCRC treatment modalities might influence the observed effects rather than merely confounding them. Furthermore, our multivariate analyses incorporated key covariates known to affect mortality risk in the mCRC population.

Nevertheless, our study has a few limitations. Its single-institution design may limit the generalizability of our findings to broader, more diverse patient populations. Additionally, the study’s retrospective nature raises the potential for selection bias. Furthermore, the pyrosequencing method was confined to detecting mutations in codons 12, 13, and 61 of *KRAS* and *NRAS*, as well as codon 600 of *BRAF*, which means we did not account for mutations outside these specific regions. Pyrosequencing also has a sensitivity threshold of approximately 10% mutant alleles. The relatively short follow-up period was also shorter than the typical median survival time for mCRC (30 months). This shorter follow-up period might have influenced the observed survival rates, potentially leading to an overestimation of survival due to the impact of censoring on the analysis. The early censoring observed in the survival curves was primarily attributed to factors such as loss to follow-up and transitions of care, where patients either discontinued follow-up within our system or sought care elsewhere. These forms of censoring are considered non-informative, meaning they are unlikely to be directly associated with the patients’ prognosis or survival outcomes. Non-informative censoring assumes that the reasons for censoring are unrelated to the underlying risk of the event being studied (death) and therefore do not introduce systematic bias into the hazard estimates [52]. However, we acknowledge that non-informative censoring still reduces the sample size over time, which may limit the robustness of the survival analysis, particularly for later time points. In addition, treatment heterogeneity posed a limitation to the study, as patients received a range of treatment modalities that might have had varying impacts on their prognosis. While we accounted for several potential confounders in our multivariable models, the influence of unmeasured factors, such as other genetic mutations or residual confounding, could not be entirely excluded. These factors might have still affected the results and highlighted the complexity of accurately assessing prognosis in this patient population. Given the aforementioned limitations, our conclusions should be interpreted cautiously.

## 5. Conclusions

In conclusion, this study identifies significant associations between specific hot-spot mutations within *KRAS* and *BRAF* genes with an increased risk of all-cause mortality in patients with mCRC. The *KRAS* hot-spot mutations c.35G>T (p.Gly12Val), c.34G>T (p.Gly12Cys), and c.38G>A (p.Gly13Asp) were associated with higher all-cause mortality risk, even among those receiving treatment for their mCRC. These findings caution against combining *KRAS* mutations from various exons/codons in biomarker assessments and clinical trial frameworks. In addition, this study underscores the necessity for vigilant follow-up in patients harboring these specific *KRAS* hot-spot mutations. In contrast, the association of the *BRAF* c.1799T>A (p.Val600Glu) mutation with all-cause mortality risk diminished in the treated mCRC subgroup, suggesting potential variability in the treatment response. These findings suggest the potential prognostic value of *KRAS* and *BRAF* mutational profiling in mCRC management. Before these findings can be integrated into clinical practice, they need validation through larger, multicenter prospective studies. Without this, their prognostic value remains uncertain. It is crucial to distinguish which findings hold immediate clinical utility and which ones are still speculative and need further investigation. Future research must also account for the interaction between genetic mutations and treatment modalities. Understanding how variations in therapeutic approaches may influence the prognostic significance of *KRAS* and *BRAF* mutations is essential for refining the mCRC risk stratification and improving patient outcomes.

## Figures and Tables

**Table 1:** *KRAS*, *NRAS*, and *BRAF* hot-spot mutations in patients with mCRC (*n* = 494).

Gene	Exon	Codon	Protein Change	Nucleotide Change	Frequency (%)
NM_004985.5 (*KRAS*)	2/3	12/13/61	*KRAS* wildtype	N/A	290 (58.2)
	2	12	p.Gly12Asp (G12D)	c.35G>A	74 (14.9)
	2	12	p.Gly12Val (G12V)	c.35G>T	51 (10.3)
	2	12	p.Gly12Cys (G12C)	c.34G>T	19 (3.9)
	2	12	p.Gly12Ser (G12S)	c.34G>A	9 (1.7)
	2	12	p.Gly12Ala (G12A)	c.35G>C	3 (0.6)
	2	12	p.Gly12Arg (G12R)	c.34G>C	3 (0.6)
	2	13	p.Gly13Asp (G13D)	c.38G>A	38 (7.7)
	3	61	p.Gln61His (Q61H)	c.183A>T	7 (1.4)
	3	61	p.Gln61Arg (Q61R)	c.182A>G	4 (0.7)
NM_002524.5 *(NRAS)*	2/3	12, 13, 61	*NRAS* wildtype	N/A	452 (91.5)
	2	12	p.Gly12Asp (G12D)	c.35G>A	5 (1.0)
	2	12	p.Gly12Val (G12V)	c.35G>T	2 (0.4)
	2	12	p.Gly12Cys (G12C)	c.34G>T	1 (0.2)
	2	12	p.Gly12Ser (G12S)	c.34G>A	1 (0.2)
	2	13	p.Gly13Val (G13V)	c.38G>T	2 (0.4)
	3	61	p.Gln61Lys (Q61K)	c.181C>A	9 (1.8)
	3	61	p.Gln61Arg (Q61R)	c.182A>G	3 (0.6)
	3	61	p.Gln61His (Q61H)	c.183A>T	1 (0.2)
	N/A	N/A	Unknown	N/A	18 (3.7)
NM_004333.6 *(BRAF)*	15	600	*BRAF* wildtype	N/A	455 (92.1)
	15	600	p.Val600Glu (V600E)	c.1799T>A	34 (6.9)
	N/A	N/A	Unknown	N/A	5 (1.0)

Abbreviations: *BRAF*, v-raf murine sarcoma viral oncogene homolog B1; *KRAS*, Kirsten rat sarcoma viral oncogene homolog; M, distant metastasis; *NRAS*, neuroblastoma RAS viral oncogene homolog.

**Table 2:** Baseline characteristics of patients with mCRC according to the hot-spot status of the *KRAS*, *NRAS*, and *BRAF* genes (*n* = 494).

Characteristics	N (%)					
	Total	*KRAS/NRAS/BRAF* Genes Hot-Spot Status				
		Wildtype 235 (47.5)	*KRAS* Mutation 202 (40.9)	*NRAS* Mutation 23 (4.7)	*BRAF* Mutation 34 (6.9)	*p*-Value
Age Mean ± SD Median, IQR						<0.0001 *
	61.0 ± 12.8	60.5 11.4	59.9 13.5	61.7 15.6	70.9 11.15	
	61.0, 16	61.0, 15	61.0, 15	65.0, 30	72.0, 14	
Sex Male Female						0.0074 *
	334 (67.6)	175 (74.5)	128 63.4)	14 (60.9)	17 (50.0)	
	160 (32.4)	60 (25.5)	74 (36.6)	9 (39.1)	17 (50.0)	
Race/ethnicity ^a^ White Hispanic Black Other						0.0426 *
	272 (55.0)	130 (55.3)	99 (49.0)	17 (73.9)	26 (76.5)	
	104 (21.1)	51 (21.7)	45 (22.3)	5 (21.7)	3 (8.8)	
	108 (21.9)	47 (20.0)	55 (27.2)	1 (4.4)	5 (14.7)	
	10 (2.0)	7 (3.0)	3 (1.5)	0 (0.0)	0 (0.0)	
Primary tumor site Right colon Transverse colon Left colon Rectum						<0.0001 *
	151 (30.6)	47 (20.0)	77 (38.1)	4 (17.4)	23 (67.7)	
	27 (5.5)	12 (5.1)	11 (5.5)	1 (4.3)	3 (8.8)	
	173 (35.0)	92 (39.2)	63 (31.2)	12 (52.2)	6 (17.7)	
	143 (28.9)	84 (35.7)	51 (25.2)	6 (26.1)	2 (5.8)	
DNA MMR ^a^ Proficient Deficient Unknown						<0.0001 *
	447 (90.5)	221 (94.0)	190 (94.1)	23 (100.0)	13 (38.2)	
	41 (8.3)	13 (5.5)	8 (3.9)	0 (0.0)	20 (58.8)	
	6 (1.2)	1 (0.5)	4 (2.0)	0 (0.0)	1 (3.0)	
Tumor grade G1 G2 G3						<0.0001 *
	118 (24.0)	55 (23.4)	48 (23.8)	8 (34.8)	7 (20.6)	
	336 (68.0)	161 (68.5)	145 (71.8)	14 (60.9)	16 (47.1)	
	40 (8.0)	19 (8.1)	9 (4.4)	1 (4.3)	11 (32.3)	
Histomorphology ^a^ Adenocarcinoma Mucinous type Signet ring cell						0.0005 *
	439 (88.9)	213 (90.6)	183 (90.6)	22 (95.7)	21 (61.8)	
	45 (9.1)	17 (7.2)	15 (7.4)	1 (4.3)	12 (35.3)	
	10 (2.0)	5 (2.2)	4 (2.0)	0 (0.0)	1 (2.9)	
Lymph node metastasis Absent Present						0.2983
	114 (23.1)	46 (19.6)	56 (27.7)	6 (26.1)	6 (17.6)	
	380 (76.9)	189 (80.4)	146 (72.3)	17 (73.9)	28 (82.4)	
Distant organ metastasis Absent Synchronous Metachronous						0.0102 *
	195 (39.5)	107 (45.5)	60 (29.7)	10 (43.5)	18 (52.9)	
	223 (45.1)	100 (42.6)	101 (50.0)	10 (43.5)	12 (35.3)	
	76 (15.4)	28 (11.9)	41 (20.3)	3 (13.0)	4 (11.8)	
Number of distant metastasis Absent One organ Two organs Three or more organs						0.06449
	195 (39.5)	107 (45.5)	60 (29.7)	10 (43.5)	18 (52.9)	
	215 (43.5)	91 (38.7)	102 (50.5)	10 (43.5)	12 (35.3)	
	61 (12.3)	25 (10.7)	31 (15.4)	1 (4.3)	4 (11.8)	
	23 (4.7)	12 (5.1)	9 (4.4)	2 (8.7)	0 (0.0)	
Liver metastasis Absent Present						0.0225 *
	310 (62.8)	156 (66.4)	113 (55.9)	14 (60.9)	27 (79.4)	
	184 (37.2)	79 (33.6)	89 (44.1)	9 (39.1)	7 (20.6)	
Lung metastasis Absent Present						0.1147
	415 (84.0)	200 (85.1)	162 (80.2)	21 (91.3)	32 (94.1)	
	79 (16.0)	35 (14.9)	40 (19.8)	2 (8.7)	2 (5.9)	
Familial risk No Yes						0.7664
	287 (58.1)	140 (59.6)	113 (55.9)	15 (65.2)	19 (55.9)	
	207 (41.9)	95 (40.4)	89 (44.1)	8 (34.8)	15 (44.1)	
Tobacco use No Yes						0.0907
	177 (35.8)	72 (30.6)	85 (42.1)	9 (39.1)	11 (32.4)	
	317 (64.2)	163 (69.4)	117 (57.9)	14 (60.9)	23 (67.6)	
Comorbidities 0 1 or 2 3 or more						0.1224
	97 (19.6)	51 (21.7)	40 (19.8)	3 (13.0)	3 (8.8)	
	224 (45.4)	110 (46.8)	93 (46.0)	8 (34.8)	13 (38.2)	
	173 (35.0)	74 (31.5)	69 (34.2)	12 (52.2)	18 (52.9)	
Anemia ^a^ No Yes						0.7366
	191 (38.7)	93 (38.6)	80 (39.6)	7 (30.4)	11 (32.4)	
	303 (61.3)	142 (60.4)	122 (60.4)	16 (69.6)	23 (67.6)	
NLR ^a^ Normal Mild stress Moderate stress Severe stress						0.1489
	235 (47.3)	114 (48.6)	91 (45.1)	6 (26.1)	24 (70.6)	
	175 (35.4)	80 (34.0)	73 (36.1)	14 (60.9)	8 (23.5)	
	65 (13.2)	33 (14.0)	28 (13.9)	2 (8.7)	2 (4.9)	
	19 (4.1)	8 (3.4)	10 (4.9)	1 (4.3)	0 (0.0)	
CEA Normal High Unknown						0.0049 *
	133 (26.9)	79 (33.6)	40 (19.8)	4 (17.4)	10 (29.4)	
	325 (65.8)	142 (60.4)	149 (73.8)	15 (65.2)	19 (55.9)	
	36 (7.3)	14 (5.0)	13 (6.4)	4 (17.4)	5 (14.7)	
Curative surgery No Yes						0.3349
	190 (38.5)	87 (37.0)	85 (42.1)	9 (39.1)	9 (26.5)	
	304 (61.5)	148 (63.0)	117 (57.9)	14 (60.9)	25 (73.5)	
Chemotherapy No Yes						0.1734
	149 (30.2)	68 (28.9)	58 (28.7)	7 (30.4)	16 (47.1)	
	345 (69.8)	167 (71.1)	144 (71.3)	16 (69.6)	18 (52.9)	
Radiotherapy No Yes						0.0036 *
	404 (81.8)	180 (76.6)	172 (85.2)	18 (78.3)	34 (100.0)	
	90 (18.2)	55 (23.4)	30 (14.8)	5 (21.7)	0 (0.0)	
Mortality rate	146 (29.6)	53 (22.6)	70 (34.7)	8 (34.8)	15 (44.1)	0.0077 *
Median OS (months) ^b^	59.7	not reached	48.1	48.9	42.7	0.0042 *

Abbreviations: *BRAF*, v-raf murine sarcoma viral oncogene homolog B1; CEA, carcinoembryonic antigen; IQR, interquartile range; *KRAS*, Kirsten rat sarcoma viral oncogene homolog; MMR, mismatch repair; NLR, neutrophil–lymphocyte ratio; *NRAS*, neuroblastoma RAS viral oncogene homolog; OS, overall survival; SD, standard deviation. ^a^ The *p*-value of the Monte Carlo Exact test. ^b^ The *p*-value of the log-rank test. * Statistical significance at the *p*-value < 0.05 level.

## Data Availability

The data presented in this study are available upon reasonable request from the corresponding author. The data are not publicly available due to patient confidentiality restrictions.

## Supplementary Materials

The following supporting information can be downloaded at https://www.mdpi.com/article/10.3390/diagnostics15050590/s1: Table S1. The study’s variables and attributes. Table S2. Baseline characteristics of patients with lymph node and/or distant metastasis CRC according to the *KRAS* gene hot-spot status. Table S3. Baseline characteristics of patients with lymph node and/or distant metastasis CRC according to the *NRAS* gene hot-spot status. Table S4. Baseline characteristics of patients with lymph node and/or distant metastasis CRC according to the *BRAF* gene hot-spot status. Figure S1. Multivariable Cox proportional hazards models for the association between the *KRAS*, *NRAF*, and *BRAF* hot-spot mutations and the risk of all-cause mortality in patients with mCRC. Figure S2. Multivariable Cox proportional hazards models for the association between specific *KRAS*, *NRAF*, and *BRAF* hot-spot mutations and the risk of all-cause mortality in patients with mCRC.
