# Supplementary material for: Mortality Outcome Associated with Specific KRAS, NRAS, and BRAF Hot-Spot Mutations in Metastatic Colorectal Cancer Patients: A Retrospective Cohort Study
Source: Diagnostics (Basel). 2025 Feb 28;15(5):590. doi: 10.3390/diagnostics15050590 (PMC11899597; doi:10.3390/diagnostics15050590)
Supplement: Supplementary file 1 [file diagnostics-15-00590-s001.zip › diagnostics-3465638-supplementary.pdf]

Supplementary Materials

Article

Mortality outcome associated with specific *KRAS*, *NRAS*, and *BRAF* hot-spot mutations in metastatic colorectal cancer patients: A retrospective cohort study

Omer Abdelgadir [1], Yong-Fang Kuo [2], M. Firoze Khan [3], Anthony O. Okorodudu [3], Yu-Wei Cheng [4] and Jianli Dong [3,*]

[1] Graduate School of Biomedical Science, University of Texas Medical Branch, Galveston, TX 77555, USA
[2] School of Public and Population Health, University of Texas Medical Branch, Galveston, TX 77555, USA ; yokuo@utmb.edu
[3] Department of Pathology, University of Texas Medical Branch, Galveston, TX 77555, USA; mfkhan@utmb.edu (M.F.K); aookorod@utmb.edu (A.O.O);
[4] Department of Laboratory Medicine, Cleveland Clinic, Cleveland, OH 44195, USA; chengy@ccf.org
* Correspondence: jidong@utmb.edu

Supplemental Materials List

- **Table S1.** The study's variables and attributes.
- **Table S2.** Baseline characteristics of patients with lymph node and/or distant metastasis CRC according to the *KRAS* gene hot-spot status.
- **Table S3.** Baseline characteristics of patients with lymph node and/or distant metastasis CRC according to the *NRAS* gene hot-spot status.
- **Table S4.** Baseline characteristics of patients with lymph node and/or distant metastasis CRC according to the *BRAF* gene hot-spot status.
- **Figure S1.** Multivariable Cox proportional hazards models for the association between the *KRAS*, *NRAF*, and *BRAF* hot-spot mutations and the risk of all-cause mortality in patients with mCRC.
- **Figure S2.** Multi-variable Cox proportional hazards models for the association between specific *KRAS*, *NRAF*, and *BRAF* hot-spot mutations and the risk of all-cause mortality in patients with mCRC.

Table S1. Study's variables and attributes.

Variable	Description	Values
NM_004985.5(*KRAS*) mutation	Kirsten rat sarcoma viral oncogene homolog gene. Mutation in *KRAS* genomic regions; exon 2 (codon 12 and 13), exon 3 (codon 61). Other *KRAS* mutations include p.Gly12Ser (G12S), p.Gly12Ala (G12A) and p.Gly12Arg (G12R), p.Gln61His (Q61H), p.Gln61Leu (Q61L), p.Gln61Arg (Q61R) and p.Gln61Glu (Q61E).	Wildtype<br>c.35G>A (p.Gly12Asp)<br>c.35G>T (p.Gly12Val)<br>c.34G>T (p.Gly12Cys)<br>c.38G>A (p.Gly13Asp)<br>Other mutations
NM_002524.5(*NRAS*) mutation	Neuroblastoma RAS viral (V-ras) oncogene homolog. Mutation in *NRAS* genomic regions; exon 2 (codon 12 and 13), exon 3 (codon 61). p.Gln61 mutations include p.Gln61Lys (Q61K), p.Gln61Arg (Q61R), p.Gln61His (Q61H) and p.Gln61Leu (Q61L). Other mutations include c.35G>A (p.Gly12Asp), c.35G>T (p.Gly12Val), c.34G>T (p.Gly12Cys), p.Gly12Ser (G12S) and p.Gly13Val (G13V).	Wildtype<br>p.Gln61 mutations<br>Other mutations<br>Unknown
NM_004333.6(*BRAF*) mutation	v-raf murine sarcoma viral oncogene homolog B1.	Wildtype<br>c.1799T>A (p.Val600Glu)<br>Unknown
Age at diagnosis	Patient's age in years at initial CRC diagnosis.	Numbers
Sex	Patient's biological sex.	Male<br>Female
Race/ethnicity	Self-identified race/ethnicity group.	White<br>Hispanic<br>Black<br>Other
Primary CRC sidedness	Primary tumor anatomical location. Right colon included Ileocecal valve, cecum, ascending colon, hepatic flexure. Left colon included splenic flexure, descending colon, and sigmoid.	Right colon<br>Transvers colon<br>Left colon<br>Rectum
DNA mismatch repair	Mismatch Repair (MMR). Microsatellite (genetic) instability in short nucleotide repeats. Expression of protein products of *MSH2, MSH6, MLH1* and *PMS2* genes by immunohistochemistry (IHC) assays.	MMR-proficient<br>MMR-deficient
Tumor grade	Measure of anaplasia	G1 (well differentiated)<br>G2 (moderately differentiated)<br>G3 (poorly differentiated)

Histomorphology	Histomorphologic phenotype	Adenocarcinoma<br>Mucinous adenocarcinoma<br>Signet ring carcinoma
Lymph node metastasis	Initial AJCC staging, any nearby lymph nodes involved.	Absent<br>Present
Distant organ metastasis	Synchronous: metastasis detected at the time of initial diagnosis of primary tumor.<br>Metachronous: metastasis detected more than 3 months of after diagnosis of primary tumor.	Absent<br>Synchronous<br>Metachronous
Number of distant metastasis	Number of distant organs involved in CRC distant metastasis	Absent<br>One organ<br>Two organs<br>Three or more organs
Liver metastasis	CRC has spread to the liver	Absent<br>Present
Lung metastasis	CRC has spread to the lungs	Absent<br>Present
Familial risk	Have relatives who have had CRC, polyp or any other types of cancer or gland-like growths developed on the mucous membrane that lines the large intestine.	No<br>Yes
Tobacco use	Self-reported use of tobacco product.	No<br>Yes
Comorbidities	Number of highly prevalent conditions that may influence cancer management alone or in combination with another condition Including other cancer, other metastatic cancer, CVD, CPD, CDH, MI, PVD, HTN, DM, peptic ulcer, rheumatological disease, dementia, hemiplegia and paraplegia, liver disease and renal disease.	0<br>1 - 2<br>3 or more
Anemia	Established diagnosis anemia at admission (pre-operative)	No<br>Yes
NLR	Relative Neutrophil-to-Lymphocyte ratio at admission (pre-operative)	Normal (1 – 3)<br>Mild stress (4 – 8)<br>Moderate stress(9 – 18)<br>Severe stress (>18)
CEA	Serum Carcinoembryonic antigen interpretation at admission (Normal for Smoker: 0.0 - 5.5, and Non-Smoker: 0.0 - 3.8) (pre-operative)	Normal<br>High<br>Unknown
Curative surgery	Patient undergone curative surgery for CRC	No<br>Yes
Chemotherapy	Patient received chemotherapy for CRC	No<br>Yes

Radiotherapy	Patient undergone radiotherapy for CRC	No<br>Yes
Survival time	Survival time in months from the date of initial CRC diagnosis to either the date of death (event) or the last follow-up date (right-censored). The last follow-up date is the last encounter as indicated in the patient's chart or study end date (July 31, 2024), whichever comes first. Patients who remained alive or died after the study's end date were right-censored (those who didn't experience the event until study ends).	Numbers

Table S2. Baseline characteristics of patients with lymph node and/or distant metastasis CRC according to the *KRAS* gene hot-spot status (*n*=494).

Characteristics	N (%)						
	NM_004985.5(*KRAS*) gene hot-spot status						
	**Wildtype** **290 (58.7)**	**c.35G>A (p.Gly12Asp) 74 (14.9)**	**c.35G>T (p.Gly12Val) 51 (10.3)**	**c.34G>T (p.Gly12Cys) 19 (3.9)**	**c.38G>A (p.Gly13Asp) 38 (7.7)**	**Other mutations 22 (4.5)**	**P-value**
Age							0.0505
• Mean ± SD	62.1 ±12.1	58.9 ±14.5	61.7 ±13.6	57.7 ±9.9	56.6 ±14.5	63.5 ±9.4	
• Median, IQR	62.0, 16	60.0, 18	61.0, 18	63.0, 13	55.0, 17	63.5, 13	
Sex							0.3943
• Male	206 (71.0)	46 (62.2)	32 (62.6)	12 (63.2)	22 (57.9)	16 (72.7)	
• Female	84 (29.0)	28 (37.8)	19 (37.4)	7 (36.8)	16 (42.1)	6 (27.3)	
Race/ethnicity [a]							0.1706
• White	171 (59.0)	34 (45.9)	26 (51.0)	8 (42.1)	21 (55.2)	12 (54.5)	
• Hispanic	61 (21.0)	21 (28.4)	9 (17.7)	2 (10.5)	8 (21.1)	3 (13.6)	
• Black	51 (17.6)	19 (25.7)	15 (29.4)	9 (47.4)	8 (21.1)	6 (27.3)	
• Other	7 (2.4)	0 (0.0)	1 (1.9)	0 (0.0)	1 (2.6)	1 (4.6)	
Primary tumor site [a]							0.1688
• Right colon	75 (25.9)	26 (35.1)	20 (39.2)	3 (15.8)	17 (44.7)	10 (45.4)	
• Transverse colon	16 (5.5)	4 (5.4)	1 (1.9)	3 (15.8)	2 (5.3)	1 (4.6)	
• Left colon	108 (37.2)	23 (31.1)	19 (37.3)	5 (26.3)	12 (31.6)	6 (27.3)	
• Rectum	91 (31.4)	21 (28.4)	11 (21.6)	8 (42.1)	7 (18.4)	5 (22.7)	
DNA MMR [a]							0.0126*
• Proficient	255 (87.9)	68 (91.8)	50 (98.0)	17 (89.5)	37 (97.4)	20 (90.9)	
• Deficient	33 (11.4)	5 (6.7)	0 (0.0)	0 (0.0)	1 (2.6)	2 (9.1)	
• Unknown	2 (0.7)	1 (1.4)	1 (2.0)	2 (10.5)	0 (0.0)	0 (0.0)	
Tumor grade [a]							0.0419*
• G1	69 (23.8)	14 (18.9)	16 (31.4)	6 (31.6)	7 (18.4)	6 (27.3)	
• G2	190 (65.5)	57 (77.0)	35 (68.6)	12 (63.2)	30 (79.0)	12 (54.6)	
• G3	31 (10.7)	3 (4.1)	0 (0.0)	1 (5.2)	1 (2.6)	4 (18.1)	
Histomorphology [a]							0.5728
• Adenocarcinoma	256 (88.3)	68 (91.9)	47 (92.2)	15 (78.9)	35 (92.1)	18 (81.8)	
• Mucinous type	28 (9.7)	4 (5.4)	3 (5.9)	4 (21.1)	3 (7.9)	3 (13.6)	
• Signet ring cell	6 (2.0)	2 (2.7)	1 (1.9)	0 (0.0)	0 (0.0)	1 (4.6)	
Familial risk							0.1750
• No	175 (60.3)	37 (50.0)	32 (62.7)	9 (47.4)	18 (47.4)	16 (72.7)	
• Yes	115 (39.7)	37 (50.0)	19 (37.3)	10 (52.6)	20 (52.6)	6 (27.3)	
Tobacco use							0.2626
• No	92 (31.7)	32 (43.2)	21 (41.2)	7 (36.8)	14 (36.8)	11 (50.0)	
• Yes	198 (68.3)	42 (56.8)	30 (58.8)	12 (63.2)	24 (63.2)	11 (50.0)	
Comorbidities							0.5743
• 0	56 (19.3)	16 (21.6)	8 (15.7)	1 (5.3)	12 (31.6)	4 (18.2)	
• 1 – 2	132 (45.5)	32 (43.2)	27 (52.9)	10 (52.6)	12 (31.6)	11 (50.0)	
• 3 or more	102 (35.2)	26 (35.2)	16 (31.4)	8 (42.1)	14 (36.8)	7 (31.8)	

Anemia [a]							0.6098
• No	109 (37.6)	31 (41.9)	21 (41.2)	9 (47.4)	15 (39.5)	6 (27.3)	
• Yes	181 (62.4)	43 (58.1)	30 (58.2)	10 (52.6)	23 (60.5)	16 (72.3)	
NLR [a]							0.5301
• Normal	144 (49.6)	29 (39.2)	22 (43.1)	12 (63.2)	20 (52.6)	8 (36.4)	
• Mild stress	100 (34.5)	29 (39.2)	18 (35.3)	6 (31.6)	12 (31.6)	10 (45.5)	
• Moderate stress	37 (12.8)	10 (13.5)	9 (17.7)	0 (0.0)	6 (15.8)	3 (13.6)	
• Severe stress	9 (3.1)	6 (8.1)	2 (3.9)	1 (5.2)	0 (0.0)	1 (4.5)	
CEA [a]							0.1327
• Normal	93 (32.1)	19 (25.7)	9 (17.7)	3 (15.8)	6 (15.8)	3 (13.6)	
• High	176 (60.7)	51 (68.9)	39 (76.5)	14 (73.7)	27 (71.1)	18 (81.8)	
• Unknown	21 (7.2)	4 (5.4)	3 (5.8)	2 (10.5)	5 (13.1)	1 (4.6)	
Curative surgery							0.8568
• No	106 (36.5)	33 (44.5)	19 (37.3)	7 (36.8)	16 (42.1)	9 (40.9)	
• Yes	184 (63.5)	41 (55.5)	32 (62.7)	12 (63.2)	22 (57.9)	13 (59.1)	
Chemotherapy							0.2583
• No	92 (31.7)	14 (18.9)	16 (31.4)	8 (42.1)	11 (28.9)	8 (36.4)	
• Yes	198 (68.3)	60 (81.1)	35 (68.6)	11 (57.9)	27 (71.1)	14 (63.6)	
Radiotherapy							0.6527
• No	232 (80.0)	62 (83.8)	44 (86.3)	14 (73.7)	33 (86.8)	19 (86.4)	
• Yes	58 (20.0)	12 (16.2)	7 (13.7)	5 (26.3)	5 (13.2)	3 (13.6)	
Mortality rate	77 (26.6)	22 (29.7)	18 (35.3)	9 (47.4)	10 (26.3)	10 (45.5)	0.1602
Median OS (months) [b]	not reached	69.8	53.0	38.9	not reached	40.4	0.1820

Abbreviation: *KRAS*, Kirsten rat sarcoma viral oncogene homolog; SD; standard deviation; IQR, interquartile range; OS, overall survival; MMR, mismatch repair; NLR, neutrophil lymphocyte ratio; CEA, carcinoembryonic antigen. Other *KRAS* mutations include p.Gly12Ser (G12S), p.Gly12Ala (G12A), .Gly12Arg (G12R), p.Gln61His (Q61H), p.Gln61Leu (Q61L), p.Gln61Arg (Q61R) and p.Gln61Glu (Q61E). [a] The p-value of Monte Carlo Exact test. [b] The p-value of log-rank test. *Statistical significance at the p-value < 0.05 level.

Table S3. Baseline characteristics of patients with lymph node and/or distant metastasis CRC according to the *NRAS* gene hot-spot status (*n*=494).

Characteristics	N (%)				
	**NM_002524.*5(NRAS)* gene hot-spot status**				
	**Wildtype 452 (91.5)**	**p.Gln61 mutation 13 (2.6)**	**Other mutations 11 (2.2)**	**Unknown 18 (3.7)**	**P-value**
Age					0.2615
• Mean ± SD	60.8 ±12.6	63.8 ±16.6	58.4 ±13.9	66.1 ±12.9	
• Median, IQR	61.0, 16	67.0, 19	53.0, 25	63.5, 15	
Sex [a]					0.9193
• Male	307 (67.9)	8 (61.5)	7 (63.6)	12 (66.7)	
• Female	145 (32.1)	5 (38.5)	4 (36.4)	6 (33.3)	
Race/ethnicity [a]					0.2603
• White	244 (53.9)	10 (76.9)	7 (63.6)	11 (61.1)	
• Hispanic	97 (21.5)	3 (23.2)	3 (27.3)	1 (5.6)	
• Black	102 (22.6)	0 (0.0)	1 (9.1)	5 (27.7)	
• Other	9 (2.0)	0 (0.0)	0 (0.0)	1 (5.6)	
Primary tumor site [a]					0.7782
• Right colon	141 (31.2)	3 (23.1)	1 (9.1)	6 (33.3)	
• Transverse colon	26 (5.8)	0 (0.0)	1 (9.1)	0 (0.0)	
• Left colon	154 (34.1)	6 (46.2)	6 (54.6)	7 (38.9)	
• Rectum	131 (28.9)	4 (30.7)	3 (27.2)	5 (27.8)	
DNA MMR [a]					0.0218*
• Proficient	411 (90.9)	13 (100.0)	11 (100.0)	12 (66.7)	
• Deficient	37 (8.1)	0 (0.0)	0 (0.0)	4 (22.2)	
• Unknown	4 (1.0)	0 (0.0)	0 (0.0)	2 (11.1)	
Tumor grade [a]					0.6784
• G1	104 (23.0)	5 (38.5)	4 (36.4)	5 (27.8)	
• G2	310 (68.6)	8 (61.5)	6 (54.6)	12 (66.7)	
• G3	38 (8.4)	0 (0.0)	1 (9.0)	1 (5.5)	
Histomorphology [a]					0.1064
• Adenocarcinoma	404 (89.4)	13 (100.0)	10 (90.9)	12 (66.7)	
• Mucinous type	39 (8.6)	0 (0.0)	1 (9.1)	5 (27.8)	
• Signet ring cell	9 (2.0)	0 (0.0)	0 (0.0)	1 (5.5)	
Familial risk					0.8472
• No	261 (57.7)	9 (69.2)	6 (54.6)	11 (61.1)	
• Yes	191 (42.3)	4 (30.8)	5 (45.4)	7 (38.9)	
Tobacco use [a]					0.5682
• No	164 (36.3)	4 (30.8)	5 (45.4)	4 (22.2)	
• Yes	288 (63.7)	9 (69.2)	6 (54.6)	14 (77.8)	
Comorbidities [a]					0.2078
• 0	92 (20.4)	2 (15.4)	1 (9.0)	2 (11.1)	
• 1 – 2	210 (46.5)	4 (30.8)	5 (45.5)	5 (27.8)	
• 3 or more	150 (33.1)	7 (53.8)	5 (45.5)	11 (61.1)	
Anemia [a]					0.7272

• No	178 (39.4)	3 (23.1)	4 (36.4)	6 (33.3)	
• Yes	274 (60.6)	10 (76.9)	7 (63.6)	12 (66.7)	
NLR [a]					0.2576
• Normal	223 (49.4)	3 (23.1)	3 (27.3)	6 (33.3)	
• Mild stress	152 (33.6)	8 (61.5)	7 (63.6)	8 (44.4)	
• Moderate stress	59 (13.1)	1 (7.7)	1 (9.1)	4 (22.3)	
• Severe stress	18 (3.9)	1 (7.7)	0 (0.0)	0 (0.0)	
CEA [a]					0.799
• Normal	122 (27.0)	4 (30.8)	0 (0.0)	7 (38.9)	
• High	298 (65.9)	8 (61.5)	8 (72.7)	11 (61.1)	
• Unknown	32 (7.1)	1 (7.7)	3 (27.3)	0 (0.0)	
Curative surgery					0.6308
• No	174 (38.5)	4 (30.8)	6 (54.6)	6 (33.3)	
• Yes	278 (61.5)	9 (69.2)	5 (45.4)	12 (66.7)	
Chemotherapy [a]					0.3862
• No	134 (29.7)	6 (46.2)	2 (18.2)	7 (38.9)	
• Yes	318 (70.3)	7 (53.8)	9 (81.8)	11 (61.1)	
Radiotherapy [a]					0.5024
• No	372 (82.3)	11 (84.6)	8 (72.7)	13 (72.2)	
• Yes	80 (17.7)	2 (15.4)	3 (27.3)	5 (27.8)	
Mortality rate	130 (28.8)	5 (38.5)	4 (36.4)	7 (38.9)	0.6647
Median OS (months) [b]	68.7	48.9	not reached	48.1	0.6387

Abbreviation: *NRAS*, neuroblastoma RAS viral oncogene homolog; SD; standard deviation; IQR, interquartile range; OS, overall survival; MMR, mismatch repair; NLR, neutrophil lymphocyte ratio; CEA, carcinoembryonic antigen. The *NRAS* p.Gln61 mutations include p.Gln61Lys (Q61K), p.Gln61Arg (Q61R), p.Gln61His (Q61H) and p.Gln61Leu (Q61L). Other *NRAS* mutations include c.35G>A (p.Gly12Asp), c.35G>T (p.Gly12Val), c.34G>T (p.Gly12Cys), p.Gly12Ser (G12S) and p.Gly13Val (G13V). [a] The p-value of Monte Carlo Exact test. [b] The p-value of log-rank test. *Statistical significance at the p-value < 0.05 level.

Table S4. Baseline characteristics of patients with lymph node and/or distant metastasis CRC according to the *BRAF* gene hot-spot status (*n*=494).

Characteristics	N (%)			
	**NM_004333.6*(BRAF)* gene hot-spot status**			
	**Wildtype 455 (92.1)**	**c.1799T>A (p.Val600Glu) 34 (6.9)**	**Unknown 5 (1.0)**	**P-value**
Age				<.0001*
• Mean ± SD	60.3 ±12.6	70.9 ±11.1	60.6 ±10.6	
• Median, IQR	61.0, 16	72.0, 14	61.0, 5	
Sex [a]				0.0610
• Male	314 (69.0)	17 (50.0)	3 (60.0)	
• Female	141 (31.0)	17 (50.0)	2 (40.0)	
Race/ethnicity [a]				0.0240*
• White	245 (53.9)	26 (76.5)	1 (20.0)	
• Hispanic	100 (21.9)	3 (8.8)	1 (20.0)	
• Black	101 (22.2)	5 (14.7)	2 (40.0)	
• Other	9 (2.0)	0 (0.0)	1 (20.0)	
Primary tumor site [a]				<.0001*
• Right colon	126 (27.7)	23 (67.6)	2 (40.0)	
• Transverse colon	24 (5.3)	3 (8.8)	0 (0.0)	
• Left colon	166 (36.5)	6 (17.7)	1 (20.0)	
• Rectum	139 (30.5)	2 (5.9)	2 (40.0)	
DNA MMR [a]				<.0001*
• Proficient	431 (94.7)	13 (38.2)	3 (60.0)	
• Deficient	21 (4.6)	20 (58.8)	0 (0.0)	
• Unknown	3 (0.7)	1 (3.0)	2 (40.0)	
Tumor grade [a]				<.0001*
• G1	111 (24.4)	7 (20.6)	0 (0.0)	
• G2	315 (69.2)	16 (47.0)	5 (100.0)	
• G3	29 (6.4)	11 (32.4)	0 (0.0)	
Histomorphology [a]				0.0002*
• Adenocarcinoma	414 (90.1)	21 (61.8)	4 (80.0)	
• Mucinous type	32 (7.0)	12 (35.3)	1 (20.0)	
• Signet ring cell	9 (1.9)	1 (2.9)	0 (0.0)	
Familial risk [a]				0.9513
• No	265 (58.2)	19 (55.9)	3 (60.0)	
• Yes	190 (41.8)	15 (44.1)	2 (40.0)	
Tobacco use [a]				0.9090
• No	164 (36.0)	11 (32.3)	2 (40.0)	
• Yes	291 (64.0)	23 (67.7)	3 (60.0)	
Comorbidities [a]				0.0171*
• 0	92 (20.2)	3 (8.8)	2 (40.0)	
• 1 – 2	211 (46.4)	13 (38.3)	0 (0.0)	
• 3 or more	152 (33.4)	18 (52.9)	3 (60.0)	
Anemia [a]				0.6310

• No	179 (39.4)	11 (32.4)	1 (20.0)	
• Yes	276 (60.6)	23 (67.6)	4 (80.0)	
NLR [a]				0.1598
• Normal	210 (46.2)	24 (70.6)	1 (20.0)	
• Mild stress	164 (36.0)	8 (23.5)	3 (60.0)	
• Moderate stress	62 (13.6)	2 (5.9)	1 (20.0)	
• Severe stress	19 (4.2)	0 (0.0)	0 (0.0)	
CEA [a]				0.3954
• Normal	122 (26.8)	10 (29.4)	1 (20.0)	
• High	302 (66.4)	19 (55.9)	4 (80.0)	
• Unknown	31 (6.8)	5 (14.7)	0 (0.0)	
Curative surgery [a]				0.3302
• No	179 (39.3)	9 (26.5)	2 (40.0)	
• Yes	276 (60.7)	25 (73.5)	3 (60.0)	
Chemotherapy [a]				0.0794
• No	132 (29.0)	16 (47.1)	1 (20.0)	
• Yes	323 (71.0)	18 (52.9)	4 (80.0)	
Radiotherapy [a]				0.0015*
• No	367 (80.7)	34 (100.0)	3 (60.0)	
• Yes	88 (19.3)	0 (0.0)	2 (40.0)	
Mortality rate	129 (28.4)	15 (44.1)	2 (40.0)	0.1231
Median OS (months) [b]	68.7	42.7	25.4	0.1761

Abbreviation, *BRAF,* v-raf murine sarcoma viral oncogene homolog B1homolog; SD; standard deviation; IQR, interquartile range; OS, overall survival; MMR, mismatch repair; NLR, neutrophil lymphocyte ratio; CEA, carcinoembryonic antigen. [a] The p-value of Monte Carlo Exact test. [b] The p-value of log-rank test. *Statistical significance at the p-value < 0.05 level.

Category	Patients (Deaths)	Adjusted HR (95% CI)	P-value
All mCRC cohort (n = 494)			
KRAS/NRAS/BRAF genes			
Wildtype	235 (53)	Ref	
KRAS mutation	202 (70)	1.75 (1.22 , 2.52)	0.0027*
NRAS mutation	23 (8)	1.17 (0.54 , 2.51)	0.6948
BRAF mutation	34 (15)	1.98 (1.07 , 3.65)	0.0285*
Treated mCRC cohort (n = 421)			
KRAS/NRAS/BRAF genes			
Wildtype	204 (46)	Ref	
KRAS mutation	171 (55)	1.76 (1.17 , 2.62)	0.0059*
NRAS mutation	20 (6)	1.27 (0.54 , 3.02)	0.5864
BRAF mutation	26 (10)	1.43 (0.69 , 2.93)	0.3269

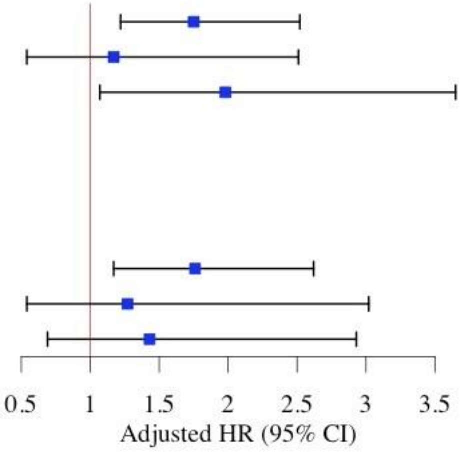

Figure S1. Multivariable Cox proportional hazards models for the association between the *KRAS*, *NRAF*, and *BRAF* hot-spot mutations and the risk of all-cause mortality in patients with mCRC. Cox model for all mCRC adjusted for age at diagnosis, comorbidities, anemia, neutrophil lymphocyte ratio, and carcinoembryonic antigen. Cox model for treated mCRC cohort adjusted for age at diagnosis, anemia, and carcinoembryonic antigen. The treated mCRC cohort included patients who received either curative surgery, chemotherapy, or radiotherapy, whether as a single treatment or in combination. Abbreviations: HR, hazard ratio; CI, confidence interval; *KRAS*, Kirsten rat sarcoma viral oncogene homolog; *NRAS*, neuroblastoma RAS viral oncogene homolog; *BRAF*, v-raf murine sarcoma viral oncogene homolog B1. *Statistical significance at the P-value < 0.05 level.

Category	Patients (Deaths)	Adjusted HR (95% CI)	P-value
All mCRC cohort (n = 494)			
NM_004985.5(KRAS) gene			
Wildtype	290 (77)	Ref	
c.35G>A (p.Gly12Asp)	74 (22)	1.21 (0.73 , 2.01)	0.4673
c.35G>T (p.Gly12Val)	51 (18)	2.01 (1.16 , 3.51)	0.0131*
c.34G>T (p.Gly12Cys)	19 (9)	2.33 (1.12 , 4.85)	0.0242*
c.38G>A (p.Gly13Asp)	38 (10)	1.43 (0.72 , 2.83)	0.3028
Other mutations	22 (10)	1.57 (0.79 , 3.13)	0.1968
NM_002524.5(NRAS) gene			
Wildtype	452 (130)	Ref	
p.Gln61 mutation	13 (5)	1.09 (0.43 , 2.78)	0.8439
Other mutations	11 (4)	1.19 (0.41 , 3.43)	0.7441
Unknown	18 (7)	0.78 (0.29 , 2.05)	0.6143
NM_004333.6(BRAF) gene			
Wildtype	455 (129)	Ref	
c.1799T>A (p.Val600Glu)	34 (15)	2.01 (1.07 , 3.78)	0.0303*
Unknown	5 (2)	1.01 (0.18 , 5.76)	0.9939
Treated mCRC cohort (n = 421)			
NM_004985.5(KRAS) gene			
Wildtype	246 (61)	Ref	
c.35G>A (p.Gly12Asp)	65 (18)	1.38 (0.75 , 2.39)	0.2523
c.35G>T (p.Gly12Val)	43 (14)	2.07 (1.12 , 3.82)	0.0201*
c.34G>T (p.Gly12Cys)	15 (7)	2.56 (1.13 , 5.81)	0.0241*
c.38G>A (p.Gly13Asp)	32 (8)	1.62 (0.76 , 3.47)	0.2113
Other mutations	20 (9)	1.96 (0.95 , 4.06)	0.0706
NM_002524.5(NRAS) gene			
Wildtype	385 (105)	Ref	
p.Gln61 mutation	11 (4)	1.46 (0.53 , 4.09)	0.4616
Other mutations	9 (2)	0.85 (0.21 , 3.61)	0.8251
Unknown	16 (6)		
NM_004333.6(BRAF) gene			
Wildtype	391 (106)	Ref	
c.1799T>A (p.Val600Glu)	26 (10)	1.28 (0.61 , 2.72)	0.5052
Unknown	4 (1)	0.52 (0.06 , 4.98)	0.5761

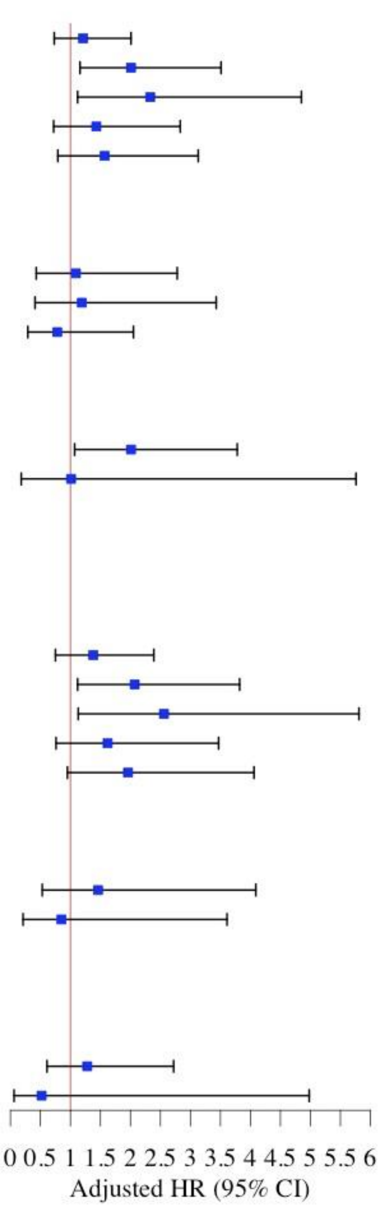

Figure S2. Multivariable Cox proportional hazards models for the association between specific *KRAS*, *NRAF*, and *BRAF* hot-spot mutations and the risk of all-cause mortality in patients with mCRC. Cox model for all mCRC adjusted for age at diagnosis, comorbidities, anemia, neutrophil lymphocyte ratio, and carcinoembryonic antigen. Cox model for treated mCRC cohort adjusted for age at diagnosis, anemia, and carcinoembryonic antigen. The treated mCRC cohort included patients who received either curative surgery, chemotherapy, or radiotherapy, whether as a single treatment or in combination. Abbreviations: HR, hazard ratio; CI, confidence interval; *KRAS*, Kirsten rat sarcoma viral oncogene homolog; *NRAS*, neuroblastoma RAS viral oncogene homolog; *BRAF*, v-raf murine sarcoma viral oncogene homolog B1. *Statistical significance at the P-value < 0.05 level.
